# Habitat Overlap of Tiger and Leopard in Banke‐Bardia Complex

**DOI:** 10.1002/ece3.72592

**Published:** 2025-12-02

**Authors:** Sagar Raj Kandel, Saroj Panthi, Bikram Shrestha, Naresh Subedi, Rabin Kadariya

**Affiliations:** ^1^ Hands for Conservation Kaski Nepal; ^2^ Division Forest Office, Baglung Ministry of Forest and Environment, Gandaki Province Pokhara Nepal; ^3^ Conservation Development Foundation (CODEFUND) Kathmandu Nepal; ^4^ Green Governance Nepal Kathmandu Nepal; ^5^ National Trust for Nature Conservation Kathmandu Nepal

**Keywords:** Banke national park, Bardia national park, habitat suitability, leopard, MaxEnt, Nepal, tiger

## Abstract

Tiger ( *Panthera tigris* ) and leopard ( *Panthera pardus* ) are two congener species and wherever they live together are found to compete for space and habitat. The study on habitat suitability and overlap between tigers and leopards in the Banke‐Bardia Complex, Nepal, aims to assess the ecological preferences and spatial distribution patterns of these big cat species within the given landscape. For the study the Banke‐Bardia Complex was considered as it is one of the priority tiger conservation units. We have gathered the GPS location of tigers and leopards from the camera trap survey. Maximum Entropy (Maxent) is software used to model the suitable habitat of species by using geo‐referenced occurrence data and environmental variables. We used area under the receiver‐operator curve (AUC) as the threshold‐independent method. We found a fair AUC for the model of leopard (0.674+/−0.043) and tiger (0.690+/− 0.012) habitat suitability model. We found 854.15 km^2^ and 867.21 km^2^ of suitable habitat for common leopard and tiger respectively throughout the study area. We identified 388.16 km^2^ of overlapping habitat between the species, which constituted 45.60% of the habitat of leopard and 44.75% of the habitat of tiger. Most of the overlapping habitat was located in the southern part of Banke National Park and western parts of Bardia National Park. The findings from this study highlight the need for inclusive conservation strategies extending beyond core protected areas. Engaging local communities in conservation efforts and promoting sustainable land‐use practices can mitigate human‐wildlife conflicts and support broader ecological integrity.

## Graphical abstract

The findings from this study have critical implications for conservation and management strategies in the Banke‐Bardia Complex. Effective conservation strategies must consider the ecological requirements of both species, address potential competition, and engage local communities in sustainable conservation practices.

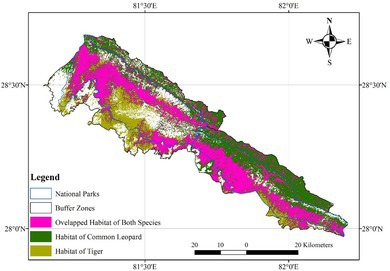

## 1 Introduction

Tiger ( *Panthera tigris* ) and leopard ( *Panthera pardus* ) are two congener species and wherever they live together are found to compete for space and habitat (Hamilton 1976; Begon et al. 1986). Large predator habitat appropriateness is conceptually defined as finding and evaluating environmental components that satisfy the specific ecological needs of the predator (Cimatti et al. 2021; Chutipong et al. 2017). This theory is based on the realization that different species have different requirements for their ideal habitats in order to thrive and procreate. Researchers assess variables such as flora type, prey quantity, and topographical features (correct predictors) to identify their habitat suitability. Therefore, the existence of a species depends on a number of variables, the most significant of which are found at various spatial scales (Bradter et al. 2013). Neglecting to take this action or incorporating the appropriate predictors at incorrect spatial scales might significantly skew the findings (Bradter et al. 2013). The theoretical framework of habitat appropriateness provides insight into the ecological niche and spatial distribution of large predators within a chosen landscape by analyzing these components (Cimatti et al. 2021; Chutipong et al. 2017).

Large predators cohabit and share common biological niches and resources within a particular landscape, and the theoretical framework pertaining to habitat overlap among them centers on the spatial intersection of their individual ranges (Harihar et al. 2011). Numerous ecological factors, such as the types of plants, the abundance of animals, and the presence of water sources, influence this phenomenon (Wisz et al. 2013; Noor et al. 2022). Its capacity to affect species relationships, population dynamics, and the general health of the ecosystem highlights the ecological significance of habitat overlap among great predators (Atkins et al. 2019).

The investigation of habitat appropriateness and tigers' and leopards' overlap is based on an understanding of the ecological mechanisms that affect these apex predators' cohabitation (Harihar et al. 2011). Big predators—typically apex predators—are essential for controlling prey numbers, which affects the composition and variety of ecosystems (Atkins et al. 2019). Overlap of habitats can result in resource competition, which can then cause behavioral changes, spatial segregation, or even outright conflicts between species (Harihar et al. 2011). Determining the habitat overlap facilitates the assessment of possible conflicts, assessment of the sustainability of prey populations, and development of successful conservation methods to preserve ecological balance by researchers and conservationists. Furthermore, habitat overlap illustrates how species are related to one another within an ecosystem. A shift in the population or behavior of a single large predator can have a domino effect on other species, affecting trophic cascades and reshaping the habitat's overall biodiversity and ecological dynamics (Atkins et al. 2019; Harihar et al. 2011). Thus, research on habitat overlap helps maintain resilient and balanced ecosystems by providing crucial insights for large predator conservation and management. Furthermore, the overlap of habitats shows how interdependent species are in an ecosystem. A single large predator's population or behavior can alter, affecting trophic cascades, affecting other species, and reshaping the habitat's overall biodiversity and ecological dynamics (Atkins et al. 2019). Therefore, research on habitat overlap provides crucial information for managing and conserving large predators, which helps to maintain resilient and balanced ecosystems.

Large predator habitat overlap is being studied because it is becoming increasingly clear how complex ecological interactions influence predator dynamics in ecosystems. Ecological study has historically frequently concentrated on individual species, but it is now more crucial to understand how various species cohabit and interact, particularly with large predators that have important places in food webs. The background work on habitat overlap has highlighted the significance of taking into account the larger ecological environment in addition to shedding light on the competitive aspects of cohabitation. The regulation of prey populations and vegetation dynamics by large predators is crucial for maintaining ecosystem stability. Therefore, studying large predators and their interactions with their habitats is an essential part of ecological research and conservation efforts. For efficient wildlife management and biodiversity conservation, it is becoming more important to look into the variables driving habitat overlap among large predators as the problems of habitat fragmentation and human‐animal conflicts worsen.

### 1.1 Global Distribution, National Status of Two Predators

Among these two sympatric carnivores, leopards have a wider range of distribution around the globe. They are known to occur from desert to mountain peaks, grassland to rocky cliffs with an upper elevation limit of 5200 m (Stein et al. 2020). Within South and Central Asia leopards are found to occur in jungles within and outside the Protected Areas (PAs) especially, in Bhutan and Nepal (Stein et al. 2020). On the contrary, tiger distribution in limited to 13 Asian countries (Goodrich et al. 2022). Both species are globally decreasing in population with a demand for conservation actions. Research and monitoring for action plans, conservation site identification, with proliferation of education regarding study focus species is the demand for the long‐term persistence of carnivores (Goodrich et al. 2022; Stein et al. 2020).

**FIGURE 1:**
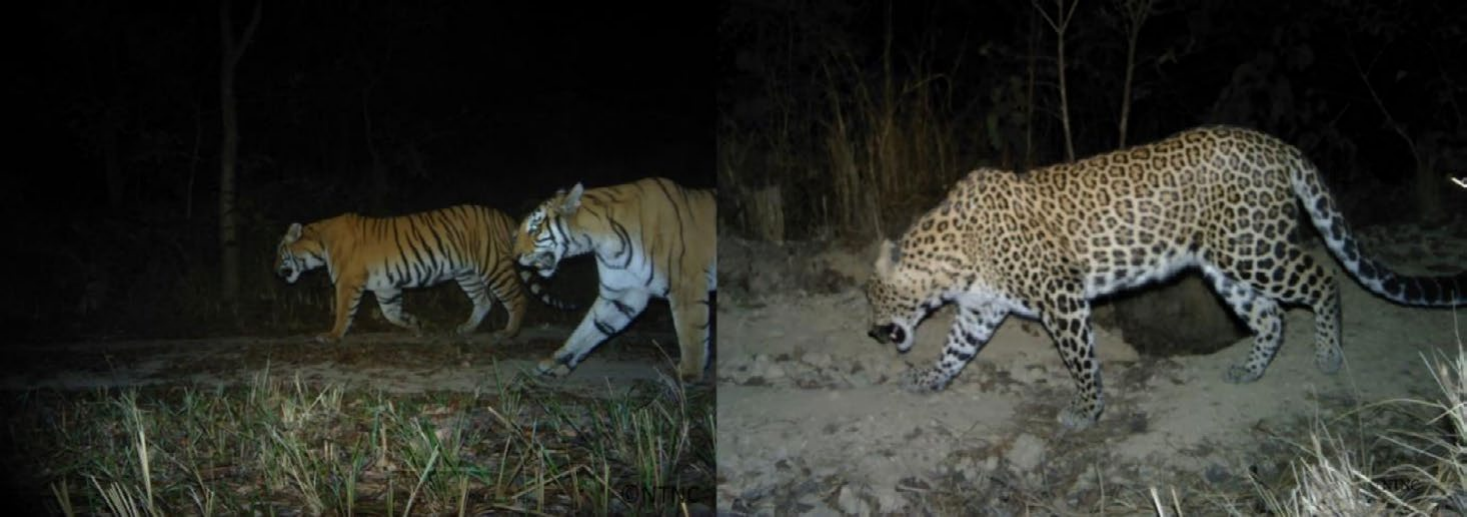
Tiger ( *Panthera tigris tigris* ) in BNP (left) and Leopard ( *Panthera pardus fusca* ) in BaNP (right) photographed during our study period.

Different forms of threats associated with target species include a decrease in wildlife prey, habitat fragmentation and loss and alteration in the niche requirements of species due to climate change (Rather et al. 2020). Under the International Union for Conservation of Nature Red List of Threatened Species (IUCN), the tiger is listed as Endangered under criteria A2abcd (Goodrich et al. 2022), and Leoaprd as Vulnerable under criteria A2cd (Stein et al. 2020). Both target species are included in Appendix S1, under the Convention on International Trade in Endangered Species of Wild Fauna and Flora (CITES), which means the species are threatened with extinction, and trade is only permitted in exceptional circumstances (CITES Secretariat 2022). Recent tiger survey reports an increase in the tiger population from 135 in 2018 to 235 in 2022, where 125 and 25 individuals were reported from Banke National Park (BaNP) and Bardia National Park (BNP) (DNPWC and DFSC 2022). Nepal has designated the tiger (Figure 1 left) under Schedule I of the National Parks and Wildlife Conservation Act of 1973. Whereas leopard (Figure 1 right) is assessed as vulnerable nationally with the assumption of fewer than 1000 mature individuals (Jnawali 2011).

In Nepal, conservation initiatives commenced in 1973 with the enactment of the National Park and Wildlife Conservation Act. Evacuation of inhabitants was undertaken to establish these protected regions, limiting public access (Bhattarai et al. 2017). Subsequently, between 1980 and 2000 ad, the Government of Nepal (GoN) promoted community involvement in conservation efforts, focusing on the expansion of protected areas and the establishment of Buffer Zones. These Buffer Zones were formally recognized in 1993 and guaranteed that 30%–50% of park revenue would be allocated for local development (Bhattarai et al. 2017). Recently, the landscape‐level conservation with participatory‐based conservation is in focus to address habitat fragmentation and change. The Government of Nepal (GoN) has directed attention towards conserving emblematic species by developing species‐specific conservation action plans for the Tiger ( *Panthera tigris tigris* ), Rhinoceros ( *Rhinoceros unicornis* ), Elephant ( *Elephas maximus* ), among others. Notably, the conservation efforts for tigers in the Terai Arc Landscape (TAL) serve as a prominent example of successful conservation initiatives.

Numerous efforts in conservation are directed towards enhancing the situation of the swiftly declining tiger *Panthera tigris* populations. Nonetheless, the potential repercussions of intra‐guild competition on other carnivores coexisting in the same area are seldom considered when devising these recovery initiatives (Harihar et al. 2011). Odden et al. (2010) have already mentioned the occurrence of interference competition restricting leopard distribution to margins of tiger territories in BNP.

**FIGURE 2:**
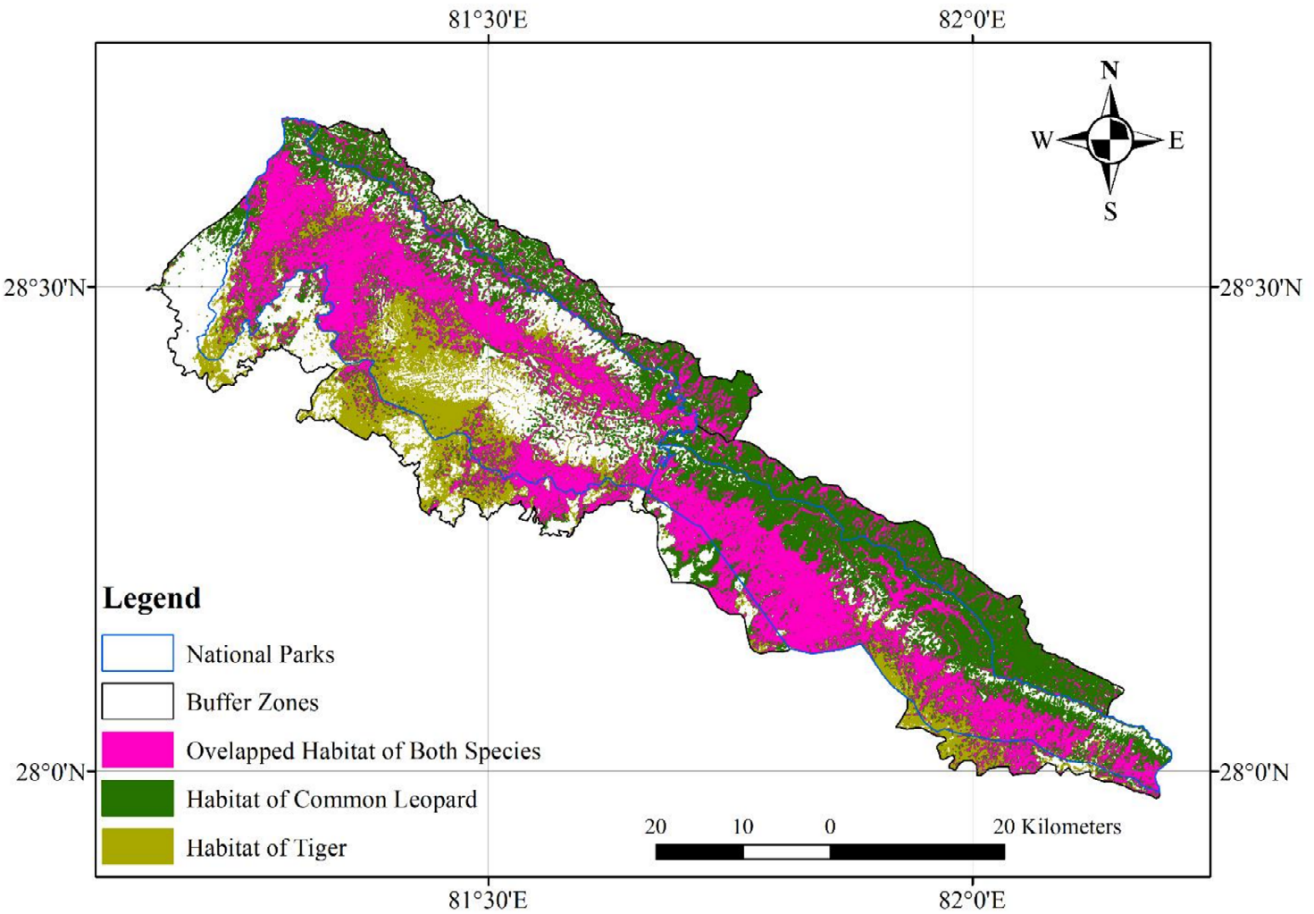
Habitat overlap between common leopard and tiger in Banke‐Bardia Complex (study area).

Our study area (Figure 2), consists of Banke and Bardia National Parks (from here called asthe Banke‐Bardia Complex) within TAL. The Banke‐Bardia Complex in Nepal stands as a unique ecological arena, hosting populations of both tigers ( *Panthera tigris* ) and leopards ( *Panthera pardus* ). Both the target species are the territorial species and required adequate prey base density for their coexistence. Much of the research has been concentrated in BNP, mainly focusing on diet, density, prey base density, occupancy, and human‐tiger conflicts. Therefore, the present study is an effort to direct the conservation of both felids in the complex by evaluating suitable habitat. An assessment of habitat is crucial for effective conservation (Kushwaha 2002). As mentioned by Creel et al. (2001), a sound approach to detecting avoidance due to interference competition among carnivores requires objective methods of mapping habitat quality for the competitors and recording their spatial distributions.

The study on habitat suitability and overlap between tigers and leopards in the Banke‐Bardia Complex, Nepal, aims to assess the ecological preferences and spatial distribution patterns of these big cat species within the given landscape. Understanding habitat suitability and the degree of overlap between these iconic big cat species is imperative for effective conservation management in this region. As human‐wildlife conflicts escalate and habitats face increasing anthropogenic pressures, a comprehensive analysis of the ecological factors shaping the coexistence of tigers and leopards becomes paramount. This manuscript delves into the intricate web of environmental variables influencing habitat suitability and explores the spatial dynamics that govern the overlap of these two carnivores in the Banke‐Bardia Complex. Through rigorous investigation and analysis, we aim to unravel the complex interactions between these predators and their habitat, providing a foundation for informed conservation strategies in this critical landscape.

## 2 Materials and Methods

### 2.1 Study Area

The study area consists of two National Parks, overall making it a Banke‐Barida Complex (as mentioned before) (Figure 2). The Banke‐Bardia Complex is the concept to foster the tiger population of Nepal. A recent tiger survey revealed 25 and 125 tigers in BaNP and BNP including in its adjoining forest areas respectively (DNPWC and DFSC 2022). Also, the overall prey density was about 33 (SE 6.6) and 90 (SE 11.2) per km^2^ in the national parks and adjoining forest areas of BaNP and BNP respectively (DNPWC and DFSC 2022). It is one of the biodiversity hotspots of Nepal. BaNP was established in 2010 with a commitment in Biodiversity conservation at the landscape level. It covers an area of 550 km^2^, with a buffer zone of 343 km^2^ covering parts of Banke, Bardia, Dang and Salyan districts within an elevation of 153–1247 m. BNP was gazetted in 1988 covering 968 km^2^, with a buffer zone of 507 km^2^ (Thapa and Chapman 2010) extending from an elevation of 150–1441 m. The BaNP is linked with a transboundary landscape that joins Suhelwa Wildlife Sanctuary in India through national and community forests towards the south (named the Kamdi corridor). It joins with BNP towards the west, which further links with Katerniaghat Wildlife Sanctuary (KWS) in India via Khata corridor, national forest and community forests. The Karnali corridor is an important corridor lying at the western edge of BNP, supporting elephant and tiger movements and the river supports highly endangered aquatic species such as the Gangetic dolphin ( *Platanista gangetica* ) and the gharial ( *Gavialis gangeticus* ).

Most of the annual precipitation occurs during 4 months of the year (late June–September). These fluctuations in climate contribute to a diverse array of ecosystems, ranging from early‐stage tall grasslands in floodplains to fully developed Sal forests at lower altitudes, and broad‐leaved forests in the Chure mountain range (MoFSC 2015). Sal (Shorea *robusta*) forest is the dominant forest cover for both national parks. Both national parks contain an array of vegetation including deciduous riverine forest, savannahs and grasslands, mixed hardwood forest, flood plain community, Bhabar and Chure Hill range (Dinerstein 1979). Nearly half of the study area lies in relatively low‐lying flat terrain of the Terai consisting of fine alluvial soil and loam, whereas, the other half consists of the Churia hills, the Himalayan foothills (MoFSC 2015).

The Banke‐Bardia complex contains at least 56 mammal species, 438 bird species, 25 reptile and amphibian species and 121 fish species (BNP 2005; Upadhyay 2005). The major predators of the area are Bengal tiger, leopard ( *Panthera pardus fusca* ), striped hyaena ( *Hyaena hyaena* ), and leopard cat ( *Prionailurus bengalensis* ). Prey species such as chital ( *Axis axis* ), hog deer ( *Axis porcinus* ), wild boar ( *Sus scrofa* ), barking deer (*Muntiacus vaginalis*), swamp deer (*Cervus duvauceli*), four‐horned antelope (*Tetracerous quadricornis*) and nilgai ( *Boselaphus tragocamelus* ) also occur in the park (Kandel 2025; Kandel et al. 2025; Wegge and Storaas 2009). Other carnivores such as sloth bear ( *Melursus ursinus* ), golden jackal ( *Canis aureus* ) and dhole ( *Cuon alpinus* ) are also present, but in low numbers (Støen and Wegge 1996; Yadav et al. 2019). Mega herbivores like one‐horned rhinoceros ( *Rhinoceros unicornis* ) and Asian elephant ( *Elephas maximus* ) are also present in the area.

Agricultural land and community forests make up the mosaic landscape of the buffer zones. An estimated 120,000 people, including indigenous Tharu people and migrants from the hill areas (Pahade), reside in the buffer zone of BNP (Thapa and Chapman 2010). Similarly, ca. 35,000 people (~5000 households) live in the buffer zone of BaNP (BaNP/NTNC‐BCP 2019).

### 2.2 Camera Trap

Banke‐Bardia complex was surveyed from December 16, 2021—March 12, 2022. The study area was divided into 4‐blocks. Camera trap study was arranged in shifting blocks because of the low number of camera traps to deploy in the field as a whole, in a 2 km × 2 km grid cell. After an extensive field survey within the (2 km × 2 km) grid cell, a pair of camera traps were installed where signs of tigers and leopards such as pugmarks, scats, scrape marks, and urination were detected along the fire lines, trails, riverbanks, and ridge lines. In BNP and the adjoining forest areas number of grid cells surveyed were 375, where in 215 grid cells tigers were captured. Whereas, in BaNP out of 344 grid cells survedyed only in 69 grid cells tigers were captured. All total in the study area total leopards capture were 128. For that Cuddeback (C1) and Panthera (V5 and V6) automated cameras were used. The cameras were programmed to take three pictures per trigger with no delay (FAP mode) using white flash. The camera traps were deployed for 15–20 nights in each of the grid cells. The images along with their metadata were retrieved on a weekly basis and stored safely for final analysis.

### 2.3 Environmental Variables

#### 2.3.1 Topographical Variables

Topographical variables such as aspect, elevation and slope have been considered in habitat modeling (Osborne et al. 2001) as related to terrestrial wildlife of Nepal (Bista et al. 2018; Panthi et al. 2019; Sharma et al. 2020). Digital Elevation Model (DEM) was downloaded from the United States Geological Survey (USGS) website (https://earthexplorer.usgs.gov/), and slope and aspect were computed from the DEM using ArcGIS software (ESRI 2017).

#### 2.3.2 Vegetation‐Related Variables

Forest cover was downloaded from the Global Forest Change (GFC) website was used as a variable (M. C. C. Hansen et al. 2013). We used the Enhanced Vegetation Index (EVI) obtained by the Moderate Resolution Imaging Spectroradiometer (MODIS). The data was then smoothed by using an adaptive Savitzky–Golay filter in the TIMESAT program (Jönsson and Eklundh 2004), the extension of ENVI was used to calculate the mean, maximum, minimum and standard deviation of the EVI and to reduce the cloud effect.

#### 2.3.3 Anthropogenic Variables

Shapfiles of buildings, paths and roads were obtained from the website of Geofabrik (https://www.geofabrik.de/data/shapefiles.html). Settlement locations were obtained from the Department of Survey, Nepal. Distance raster files of paths, roads and settlements were created by the help of ArcGIS (ESRI 2017). Land use and land cover data were downloaded from the website of the International Centre for Integrated Mountain Development (ICIMOD; http://www.icimod.org) (Uddin et al. 2015) and incorporated into the model.

### 2.4 Prediction of Suitable Habitat of Species

Maximum Entropy (MaxEnt) is a software used to model the suitable habitat of species by using geo‐referenced occurrence data and environmental variables (Phillips et al. 2006). We incorporated the variables listed in Table 1 into MaxEnt along with our occurrence data to determine habitat suitability for common leopard and tiger within our study area. The MaxEnt program is widely used to map wildlife habitat and find relationships to diverse variables (Aryal, Shrestha, et al. 2016; Aryal, Lamsal, et al. 2016; Bista et al. 2018; Dhami et al. 2023; Karki and Panthi 2021; Panthi 2018; Panthi et al. 2019, 2021; Pokharel et al. 2016; Sharma et al. 2020; Shrestha et al. 2021). Multicollinearity between environmental variables described in Table 1 is acceptable (|VIF| < 05.24) for both species. We used remove duplicated options to select only one point randomly within one pixel to lessen the spatial autocorrelation. Finally, 78 occurrence points of common leopard and 243 points of tiger were used for the modeling. We selected 1000 maximum iterations and 10 replicates during modeling (Barbet‐Massin et al. 2012). Our study area covers only two national parks, so we used 1000 background points.

**TABLE 1:** Anthropogenic variables and environmental variables incorporated to determine the suitable habitat of target species for the present study.

Category	Source	Variable	Type	Unit
Topographic	USGS	Elevation	Continuous	m
		Slope	Continuous	Degree
		Aspect	Continuous	Degree
	GEOFABRIK	Distance to water	Continuous	m
Vegetation‐related	MODIS	Annual mean EVI	Continuous	Dimensionless
		Standard deviation of EVI	Continuous	Dimensionless
		Annual maximum EVI	Continuous	Dimensionless
		Annual minimum EVI	Continuous	Dimensionless
	GFC	Forest Cover	Continuous	Dimensionless
Anthropogenic	GEOFABRIK	Distance to settlement	Continuous	m
		Distance to building	Continuous	m
		Distance to motor road	Continuous	m
		Distance to path	Continuous	m
	International Centre for Integrated Mountain Development	Land use/land cover	Categorical	Dimensionless

The model was validated by two methods: threshold dependent and threshold independent. In the threshold dependent method we provided the threshold to maximize the sum of specificity and sensitivity but in the threshold independent method the value of accuracies was directly obtained from the model. We chose true skill statistics (TSS) as the threshold dependent method. TSS = Sensitivity + Specificity −1, and ranges from −1 to 1, where values < 0 indicate a performance no better than random and 1 indicates a perfect fit (Allouche et al. 2006). We calculated TSS for all 10 model outputs, and the final TSS was averaged from all ten replications (Bista et al. 2018; Jiang et al. 2014; Panthi 2018). We used area under the receiver‐operator curve (AUC) as the threshold independent method. An AUC < 0.7 denotes poor model performance, 0.7–0.9 denotes moderately useful model performance, and > 0.9 denotes excellent model performance (Pearce and Ferrier 2000). For species distribution models that have presence‐only data the threshold to maximize the sum of sensitivity and specificity to maximize the TSS is recommended (Liu et al. 2013) so we used this threshold to convert the continuous habitat suitability map to a presence/absence binary map.

## 3 Results

### 3.1 Habitat of Common Leopard

We found 854.15 km^2^ of suitable habitat for the common leopard throughout the study area (Table 2, Figure 2). The core zone of Banke NP and Buffer Zone (BZ) contained 311.158 km^2^ and 193.21 km^2^ of suitable habitat for the common leopard respectively. Similarly, the core zone of Bardia NP and BZ contained 237.9 km^2^ and 111.46 km^2^ of suitable habitat for the common leopard respectively.

**TABLE 2:** Suitable habitat of common leopard and tiger in study area.

S.N.	Boundary of PA	Area of suitable habitat (sq.km)		
		Leopard	Tiger	Overlapped (leopard and tiger)
1	Banke NP	311.58	236.85	154.43
2	Banke BZ	193.21	65.96	29.02
3	Bardia NP	237.9	441.95	176.68
4	Bardia BZ	111.46	122.45	28.03
Total		854.15	867.21	388.16

### 3.2 Habitat of Tiger

The study identified 867.21 km^2^ of suitable habitat for tiger throughout the study area (Table 2, Figure 2). The core zone of Banke NP and BZ contained 236.85 km^2^ and 65.96 km^2^ of suitable habitat for the tiger respectively. Similarly, the core zone of Bardia NP and BZ contained 441.95 km^2^ and 122.45 km^2^ of suitable habitat for the tiger respectively.

### 3.3 Habitat Overlap Between Common Leopard and Tiger

We identified 388.16 km^2^ of overlapping habitat between the species, which constituted 45.60% of the habitat of leopard and 44.75% of the habitat of tiger. Most of the overlapping habitat was located in the southern part of Banke National Park and western parts of Bardia National Park. The overlapped habitat of the two species is located within a 331.11 km^2^ area located inside the core zone of the national parks and only 57.05 km^2^ area located inside the BZ of the national parks.

### 3.4 Accuracies of the Models

We found a fair AUC for the model of leopard (0.674+/−0.043) and tiger (0.690+/− 0.012) habitat suitability model. The thresholds (0.336 for common leopard and 0.386 for tiger model) to maximize the sum of sensitivity and specificity were used to calculate the TSS and to convert the continuous probabilistic map to a binary suitable/unsuitable map. TSS of the models of leopard and tiger was 0.352+/−0.069 and 0.387+/−0.028, respectively (Table 3).

**TABLE 3:** Accuracies of the models.

Common leopard			
Replicates	Threshold	AUC	TSS
0	0.400	0.668	0.306
1	0.270	0.663	0.365
2	0.250	0.595	0.237
3	0.380	0.693	0.395
4	0.310	0.719	0.398
5	0.380	0.702	0.405
6	0.360	0.747	0.475
7	0.450	0.645	0.301
8	0.300	0.657	0.299
9	0.260	0.651	0.340
Average	0.336	0.674	0.352
Std	0.068	0.043	0.069

Tiger			
Replicates	Threshold	AUC	TSS
0	0.440	0.682	0.356
1	0.330	0.667	0.351
2	0.380	0.694	0.412
3	0.320	0.683	0.387
4	0.280	0.684	0.388
5	0.430	0.705	0.443
6	0.410	0.703	0.409
7	0.370	0.701	0.385
8	0.410	0.681	0.368
9	0.490	0.698	0.370
Average	0.386	0.690	0.387
Std	0.063	0.012	0.028

## 4 Discussion

### 4.1 Habitat Suitability and Overlap

The present study identifies the habitat suitability of two large carnivores in Banke and Bardia National Parks including their buffer zones using the MaxEnt modeling. Our study identified 854.15 km^2^ of suitable habitat for common leopards and 867.21 km^2^ for tigers in the Banke‐Bardia Complex. This indicates a substantial degree of habitat suitability for both species within this region, underscoring the complex interplay of ecological variables that support the coexistence of these apex predators. The overlapping habitat of 388.16 km^2^, constituting 45.60% of the leopard's habitat and 44.75% of the tiger's habitat, highlights significant spatial intersection, particularly in the southern part of Banke National Park and the western part of Bardia National Park. This substantial overlap is indicative of shared ecological niches and resources, which have implications for interspecies interactions and conservation strategies.

The substantial degree of habitat suitability for both species within this region is intrinsically linked to their population densities. The recent tiger survey revealed 25 tigers in BaNP and 125 tigers in BNP, indicating relatively high tiger densities in these areas (DNPWC and DFSC 2022). Similarly, the presence of common leopards throughout the complex, as evidenced by their occupancy and the prey base densities, suggests that the suitable habitats identified are capable of supporting considerable populations of both predators.

### 4.2 Ecological Implications of Habitat Overlap

The significant overlap between leopard and tiger habitats suggests potential for both competition and coexistence. Previous studies have noted that such overlap can lead to interference competition, where the dominant tigers may restrict leopards to the peripheries of their range (Odden et al. 2010). This spatial segregation likely reduces direct competition for resources such as prey and territory.

In our study, leopards showed a broader habitat use extending from the core zones inside the national park to the surrounding fringe areas, with a relatively higher preference towards the fringes (Figure 2). This pattern aligns with other findings that predict suitable leopard habitats both inside and outside protected areas (Malla et al. 2023). Leopards' adaptability and their higher abundance near human settlements (Gupta et al. 2021) explain their occurrence in peripheral zones. Tigers, on the other hand, primarily utilize core areas, which could be due to interference and inter‐guild competition. Leopards tend to avoid the tigers (Kafley et al. 2019), and often use areas extensively grazed by livestock. Increased human activities like cattle grazing restrict tigers to core areas (Smith et al. 1998). BZ are considered marginal habitats with significant livestock disturbances. Human disturbance negatively impacts tiger occupancy (Kafley et al. 2019), while leopards show higher tolerance to human activities (Athreya et al. 2013), explaining their habitat suitability around BZ in our studied areas. Despite this, most of the spatial overlap between tigers and leopards occurs in the core zones, where key resources like prey, dense forest cover, and low human disturbance are concentrated and occasionally shared by both species.

The core zones of the national parks contained the majority of the overlapping habitats (331.11 km^2^), highlighting the importance of protected areas in supporting biodiversity and predator coexistence. These core zones provide critical resources and environmental conditions essential for both species' survival. The overlap in these protected zones indicates effectiveness in maintaining the ecological balance necessary for sustaining high predator densities.

In both national parks, large areas of suitable habitat for both tigers and leopards were found within the core zones, primarily forested areas with dense canopy and minimum disturbance, reflecting high habitat quality. Tiger habitat suitability was higher in the core areas, with 236.85 km^2^ in Banke and 441.95 km^2^ in Bardia. Along the marginal areas, the ratio of good habitat quality was lower, with tigers using these areas occasionally (Landscape analysis of tiger). In Chitwan NP, Nepal, tigers occupy the core habitats, including grassland and riverine forest, while leopards use Sal Forest and marginal areas (Lamichhane et al. 2019).

### 4.3 Conservation and Management Implications

The findings from this study have critical implications for conservation and management strategies in the Banke‐Bardia Complex. Firstly, the overlap of habitats within the core zones of national parks underscores the need for stringent protection and management of these areas to ensure the sustainability of both predator populations. These core zones provide essential resources and environmental conditions conducive to the survival of both species. With increasing tiger populations in Bardia (88) and Banke (23) NPs (DNPWC and DFSC 2022), it is crucial to note that tiger habitat is closely related to prey abundance (Smith et al. 2001; Kafley et al. 2016). Current prey abundance and park sizes are insufficient (Aryal, Shrestha, et al. 2016; Aryal, Lamsal, et al. 2016), making it challenging to maintain these felid populations and manage human–wildlife conflicts (Sijapati et al. 2021).

Secondly, the significant habitat overlap in buffer zones (57.05 km^2^) highlights the need for inclusive conservation strategies extending beyond core protected areas. Engaging local communities in conservation efforts and promoting sustainable land‐use practices can mitigate human‐wildlife conflicts and support broader ecological integrity (Shrestha et al. 2022). Given that buffer zones often interface with human settlements and agricultural lands, community‐based conservation initiatives and education programs are essential to balance wildlife conservation with local livelihoods. Leopards, which frequently roam the peripheries of the park and are more involved in conflicts (Sijapati et al. 2021; Athreya et al. 2013), underscore the importance of maintaining connectivity and mitigating human‐felid conflicts.

## 5 Conclusion

The Banke‐Bardia Complex serves as a critical landscape for the conservation of both tigers and leopards, offering suitable habitats that support their ecological needs. The substantial habitat overlap observed in this study highlights the intricate dynamics of predator coexistence and the importance of protected areas in maintaining biodiversity. Effective conservation strategies must consider the ecological requirements of both species, address potential competition, and engage local communities in sustainable conservation practices. By fostering a holistic approach to wildlife management, we can ensure the long‐term persistence of these iconic predators and the ecological balance of the Banke‐Bardia Complex.

The use of MaxEnt modeling in this study provided a robust framework for assessing habitat suitability and overlap. The models exhibited fair performance with AUC values of 0.674 for leopards and 0.690 for tigers, and TSS values of 0.352 and 0.387 respectively. These metrics indicate moderate predictive accuracy, suggesting that the selected environmental variables effectively captured the habitat preferences of both species. However, future research could benefit from incorporating additional variables such as prey density and human disturbance factors to further refine habitat suitability models.

While this study provides valuable insights into habitat suitability and overlap, several avenues for future research could enhance our understanding and conservation of these apex predators. Long‐term monitoring of predator populations and their prey base is crucial to detect changes in habitat use and overlap patterns over time. Additionally, investigating the behavioral responses of tigers and leopards to habitat overlap, such as changes in hunting strategies or temporal activity patterns, could shed light on their coexistence mechanisms.

## Author Contributions

**Sagar Raj Kandel:** conceptualization (equal), data curation (equal), formal analysis (equal), funding acquisition (equal), investigation (equal), methodology (equal), project administration (equal), resources (equal), software (equal), supervision (equal), validation (equal), visualization (equal), writing – original draft (equal), writing – review and editing (equal). **Saroj Panthi:** conceptualization (equal), data curation (equal), formal analysis (equal), methodology (equal), supervision (equal), validation (equal), writing – original draft (equal), writing – review and editing (equal). **Bikram Shrestha:** conceptualization (equal), data curation (equal), formal analysis (equal), methodology (equal), supervision (equal), validation (equal), visualization (equal), writing – original draft (equal), writing – review and editing (equal). **Naresh Subedi:** conceptualization (equal), data curation (equal), methodology (equal), resources (equal), supervision (equal), visualization (equal), writing – original draft (equal). **Rabin Kadariya:** conceptualization (equal), data curation (equal), methodology (equal), resources (equal), supervision (equal), writing – original draft (equal).

## Funding

This work was supported by the Rufford Foundation, 36129‐B.

## Conflicts of Interest

The authors declare no conflicts of interest.

## Supporting information


**Data S1:** ece372592‐sup‐0001‐DataS1.docx.

## Data Availability

All the required data is uploaded as Supporting Information.
