# Supplementary material for: Habitat Overlap of Tiger and Leopard in Banke‐Bardia Complex
Source: Ecol Evol. 2025 Dec 2;15(12):e72592. doi: 10.1002/ece3.72592 (PMC12670131; doi:10.1002/ece3.72592)
Supplement: Supplementary file 1 — Data S1: ece372592‐sup‐0001‐DataS1.docx. [file ECE3-15-e72592-s001.docx]

Annex

VIF calculation result

> ## VIF Calculation

> ## T.A. GROEN (GROEN@ITC.NL)

> setwd("D:\\E\\Research\\Tiger_Leopard\\Analysis")

> d<-read.csv("Leopard Sample.csv",header=TRUE)

> d

Slope Forest Evi_Max Evi_mean Evi_min Evi_sd Dist_build Dist_road Dist_path Dist_settle

1 37.205452 0 0.755906 0.120678 -0.267717 0.364590 0.140834 0.072413 0.072299 0.041164

2 8.032308 60 0.574803 -0.007874 -0.417323 0.256386 0.127603 0.103550 0.108629 0.075622

3 30.097172 27 0.795276 0.080452 -0.535433 0.340483 0.116309 0.070045 0.068429 0.056776

4 3.502482 74 1.000000 0.150291 -0.464567 0.381074 0.111422 0.067339 0.088628 0.072291

5 18.168600 78 0.519685 0.013009 -0.519685 0.271220 0.142217 0.101339 0.108407 0.077528

6 12.239436 47 0.834646 0.201301 -0.370079 0.309376 0.128863 0.090959 0.118048 0.086212

7 14.509845 74 0.559055 0.112804 -0.393701 0.271549 0.123126 0.083296 0.088153 0.065561

8 7.498459 81 0.913386 0.109209 -0.314961 0.403591 0.153778 0.104980 0.103584 0.063035

9 3.700742 44 0.748031 0.070866 -0.590551 0.387130 0.113493 0.070113 0.098367 0.062948

10 8.803688 63 0.866142 0.257617 -0.417323 0.321231 0.112505 0.066565 0.109567 0.034922

11 3.841636 54 0.905512 0.211571 0.000000 0.365645 0.136224 0.066361 0.156779 0.096533

12 13.258517 68 0.889764 0.256077 -0.078740 0.311569 0.091140 0.048398 0.117095 0.007212

13 19.870438 35 0.834646 0.153543 -0.448819 0.353315 0.120402 0.076830 0.133960 0.035713

14 19.360943 87 0.574803 0.330366 -0.251969 0.126780 0.071958 0.027673 0.087405 0.025191

15 3.707420 78 0.874016 0.081308 -0.440945 0.336348 0.169817 0.072651 0.177921 0.053221

16 21.677601 12 0.881890 0.197193 -0.385827 0.301684 0.055921 0.000000 0.057768 0.070953

17 17.675507 75 0.314961 0.009243 -0.220472 0.213096 0.045467 0.000000 0.042714 0.069901

18 22.661791 82 0.629921 0.178706 -0.543307 0.308136 0.095067 0.050124 0.130926 0.091232

19 4.035161 51 0.850394 0.159534 -0.661417 0.288721 0.047857 0.016361 0.014283 0.093578

20 15.864170 57 0.448819 0.053578 -0.070866 0.244400 0.042041 0.011256 0.021649 0.085644

21 13.551894 63 0.543307 0.071893 -0.212598 0.192234 0.029023 0.004714 0.028494 0.058291

22 29.768476 71 0.685039 -0.005135 -0.456693 0.344099 0.029918 0.013588 0.030411 0.065133

23 1.773568 63 0.496063 -0.130092 -0.362205 0.264625 0.060259 0.036141 0.115637 0.068047

24 13.088477 68 0.779528 0.251797 -0.354331 0.315754 0.083061 0.019247 0.035067 0.052453

25 11.523214 56 0.755906 0.193085 -0.212598 0.266193 0.078694 0.009844 0.053038 0.038420

26 6.223135 71 0.818898 0.186580 -0.275591 0.343749 0.056820 0.053795 0.131830 0.066944

27 3.055773 62 0.716535 0.175796 -0.472441 0.216862 0.069824 0.028295 0.039889 0.044697

28 1.745464 47 0.417323 -0.046046 -0.385827 0.232137 0.037600 0.015811 0.096285 0.045027

29 16.996269 47 0.354331 -0.053578 -0.779528 0.251096 0.012500 0.023806 0.024167 0.062569

30 9.940693 78 0.834646 0.376070 -0.062992 0.216634 0.051974 0.037616 0.027679 0.056602

31 11.536608 37 0.811024 0.074803 -0.322835 0.266182 0.032524 0.017797 0.051275 0.077402

32 27.250202 64 1.055118 0.281753 -0.559055 0.440985 0.021951 0.016835 0.048000 0.072534

33 1.278555 62 0.645669 0.235536 -0.275591 0.228016 0.038286 0.025339 0.027137 0.043725

34 2.718915 70 0.716535 0.156453 -0.511811 0.326894 0.023057 0.022507 0.025119 0.060526

35 16.235348 79 0.976378 0.239815 -0.480315 0.435448 0.040036 0.064983 0.073689 0.081617

36 15.428384 0 0.755906 0.157823 -0.881890 0.254265 0.009234 0.007011 0.070568 0.038907

37 0.195993 47 0.181102 -0.176994 -0.291339 0.236096 0.004946 0.004730 0.004045 0.051431

38 2.696062 49 1.173228 0.364772 -0.181102 0.388820 0.010957 0.004167 0.004598 0.032281

39 4.645838 58 0.645669 0.151147 -0.503937 0.294883 0.005618 0.005241 0.000278 0.037240

40 10.391327 67 0.968504 -0.049298 -0.653543 0.478939 0.025357 0.024058 0.089481 0.063637

41 12.534564 0 0.858268 0.212598 -0.566929 0.334450 0.046245 0.049222 0.100136 0.083910

42 3.007323 64 0.866142 0.152859 -0.385827 0.308738 0.015578 0.009546 0.009501 0.022057

43 2.721039 22 0.826772 0.006333 -0.622047 0.427274 0.011167 0.011335 0.005457 0.011948

44 3.459976 54 0.590551 0.066244 -0.472441 0.248270 0.058651 0.055862 0.060607 0.056219

45 43.147312 29 0.614173 0.286546 -0.551181 0.173494 0.026113 0.026668 0.079147 0.069569

46 3.924254 65 0.921260 0.093632 -0.614173 0.463384 0.038988 0.036198 0.041482 0.036517

47 13.376176 72 0.724409 0.256248 -0.220472 0.270454 0.074406 0.057948 0.114514 0.113648

48 12.601331 75 0.944882 0.288600 -0.472441 0.329779 0.037701 0.038156 0.065772 0.078357

49 15.832048 27 1.000000 0.406539 -0.614173 0.286204 0.085314 0.045347 0.120341 0.119487

50 1.279499 56 0.748031 0.021739 -0.629921 0.328188 0.018568 0.008629 0.011027 0.024058

51 0.543545 47 0.881890 0.329682 -0.566929 0.443446 0.025059 0.012423 0.018907 0.023049

52 0.538236 54 0.850394 0.249059 -0.858268 0.392275 0.057538 0.051343 0.059173 0.060211

53 12.063018 56 0.960630 0.483054 -0.629921 0.266774 0.033177 0.033559 0.008958 0.051976

54 14.024535 78 0.811024 0.115371 -0.803150 0.366610 0.040783 0.039831 0.019510 0.070122

55 2.099242 55 0.779528 -0.025676 -0.440945 0.312790 0.044100 0.037260 0.063199 0.067860

56 0.715539 45 0.740157 0.279870 -0.637795 0.240943 0.024778 0.020192 0.029045 0.039621

57 1.698943 70 0.897638 0.149606 -0.677165 0.461668 0.030082 0.025738 0.050547 0.059125

58 2.951908 47 0.740157 0.228518 -0.070866 0.195798 0.018109 0.017218 0.037278 0.050462

59 0.485273 79 0.976378 0.175967 -0.842520 0.425010 0.029649 0.005457 0.084751 0.089168

60 12.430635 47 0.929134 0.187778 0.039370 0.411372 0.008718 0.005945 0.045834 0.069643

61 2.427020 58 0.488189 -0.031154 -0.716535 0.370185 0.006485 0.002373 0.033866 0.062791

62 14.389716 49 0.968504 0.300068 -0.456693 0.387771 0.072347 0.067211 0.084407 0.104012

63 8.759390 78 0.661417 0.275591 -0.700787 0.213074 0.015758 0.016972 0.057747 0.086911

64 12.587852 95 0.165354 -0.169462 -0.622047 0.163454 0.001620 0.002992 0.050205 0.081992

65 19.285009 70 0.740157 0.247860 -0.574803 0.260500 0.012796 0.018392 0.048408 0.085909

66 3.338962 0 0.889764 0.119993 -0.346457 0.442567 0.054150 0.007098 0.049017 0.062198

67 10.106950 71 0.653543 0.304519 -0.125984 0.251378 0.048769 0.047728 0.090787 0.119085

68 11.073174 95 0.842520 0.205580 -0.771654 0.371356 0.039256 0.021683 0.053359 0.075933

69 9.997787 95 0.708661 0.297672 -0.062992 0.217842 0.030895 0.032895 0.074377 0.075041

70 8.901667 89 0.527559 -0.103218 -0.448819 0.285509 0.036104 0.032530 0.084556 0.088197

71 0.946206 96 0.952756 0.174427 -0.228346 0.389061 0.060694 0.025562 0.105384 0.108327

72 12.850146 62 0.543307 0.049469 -0.496063 0.203818 0.043137 0.031252 0.060126 0.061020

73 5.212578 0 0.677165 0.168093 -0.448819 0.346199 0.070257 0.022500 0.030757 0.031112

74 20.702446 54 0.921260 0.131462 -0.527559 0.377288 0.052371 0.034252 0.050489 0.046829

75 4.983716 82 0.748031 0.201130 -0.125984 0.279168 0.076538 0.013103 0.027499 0.028514

76 4.907880 56 0.677165 -0.011640 -0.433071 0.280812 0.089447 0.003869 0.011948 0.012781

Dist_water Aspect Elevation

1 0.010647 165.963760 1135

2 0.002115 139.969742 493

3 0.007098 183.990921 1325

4 0.020050 78.690071 1187

5 0.001667 335.449554 418

6 0.001242 326.040924 333

7 0.011311 192.030594 1112

8 0.001389 221.185928 401

9 0.026010 6.709836 1206

10 0.022691 285.945404 1263

11 0.002778 191.309937 231

12 0.010323 327.994629 1353

13 0.007959 172.504150 532

14 0.010039 4.184916 1196

15 0.026117 213.690063 418

16 0.009690 187.943466 802

17 0.000000 220.072891 709

18 0.017269 276.581940 902

19 0.000278 110.224861 392

20 0.000556 70.463348 401

21 0.001964 31.908106 410

22 0.000000 293.694214 427

23 0.023057 116.565048 228

24 0.001496 301.328705 371

25 0.004722 256.263733 259

26 0.003106 202.619873 208

27 0.005840 50.194427 256

28 0.011307 81.869896 186

29 0.004176 217.736755 682

30 0.010722 53.130100 501

31 0.000556 104.300278 300

32 0.015541 179.060806 562

33 0.017325 108.434952 211

34 0.001964 74.744881 214

35 0.016342 306.347443 577

36 0.001145 57.264774 475

37 0.003143 26.565050 193

38 0.010199 315.000000 181

39 0.010787 19.983107 198

40 0.020280 26.995838 562

41 0.000278 188.615646 400

42 0.005893 12.994617 191

43 0.000786 33.690067 206

44 0.004444 54.462322 275

45 0.012022 134.646332 653

46 0.010471 315.000000 240

47 0.006443 186.842773 943

48 0.011660 144.865814 608

49 0.000000 102.264771 528

50 0.011667 251.565048 187

51 0.015156 225.000000 193

52 0.012073 326.309937 235

53 0.003977 81.469231 552

54 0.001389 302.619232 591

55 0.000556 186.340195 194

56 0.015409 11.309932 180

57 0.002357 171.869904 184

58 0.003239 74.744881 175

59 0.011867 225.000000 191

60 0.005457 3.239700 604

61 0.002833 344.427460 586

62 0.006719 176.729507 580

63 0.001242 20.376434 617

64 0.002485 2.419508 637

65 0.000393 338.574707 640

66 0.003611 161.565048 825

67 0.007082 213.690063 423

68 0.003056 18.434948 755

69 0.006334 167.619247 552

70 0.000278 247.249023 337

71 0.001779 329.036255 289

72 0.004101 310.486023 388

73 0.000000 315.000000 262

74 0.001111 297.121307 438

75 0.002485 180.000000 247

76 0.010722 300.963745 226

[ reached 'max' / getOption("max.print") -- omitted 2 rows ]

> VIFcalc<-function(d)

+ {

+ result<-data.frame(var=c(names(d)),

+ VIF=numeric(length(d[1,])))

+ for(i in (1:length(d[1,])))

+ {

+ result$VIF[i] <-1/(1-summary(lm(d[,i] ~ .,data=d[,names(d)!=names(d)[i]]))$r.squared)

+ }

+ result<-result[sort(result$VIF,decreasing=T,index.return=T)$ix,]

+ return(result)

+ }

> VIFcalc(data.frame(d$Slope,d$Forest,d$Evi_Max,d$Evi_mean,d$Evi_min,d$Evi_sd,d$Dist_build,d$Dist_road,d$Dist_path,d$Dist_settle,d$Dist_water,d$Aspect,d$Elevation,

+ ))

Error in data.frame(d$Slope, d$Forest, d$Evi_Max, d$Evi_mean, d$Evi_min, :

argument is missing, with no default

> VIFcalc(data.frame(d$Slope,d$Forest,d$Evi_Max,d$Evi_mean,d$Evi_min,d$Evi_sd,d$Dist_build,d$Dist_road,d$Dist_path,d$Dist_settle,d$Dist_water,d$Aspect,d$Elevation,

+ ))

Error in data.frame(d$Slope, d$Forest, d$Evi_Max, d$Evi_mean, d$Evi_min, :

argument is missing, with no default

> VIFcalc(data.frame(d$Slope,d$Forest,d$Evi_Max,d$Evi_mean,d$Evi_min,d$Evi_sd,d$Dist_build,d$Dist_road,d$Dist_path,d$Dist_settle,d$Dist_water,d$Aspect,d$Elevation,))

Error in data.frame(d$Slope, d$Forest, d$Evi_Max, d$Evi_mean, d$Evi_min, :

argument is missing, with no default

> VIFcalc(data.frame(d$Slope,d$Forest,d$Evi_Max,d$Evi_mean,d$Evi_min,d$Evi_sd,d$Dist_build,d$Dist_road,d$Dist_path,d$Dist_settle,d$Dist_water,d$Aspect,d$Elevation))

var VIF

3 d.Evi_Max 5.242604

7 d.Dist_build 3.681390

6 d.Evi_sd 3.592190

8 d.Dist_road 3.212680

4 d.Evi_mean 2.938526

9 d.Dist_path 2.872030

13 d.Elevation 1.862582

1 d.Slope 1.737728

10 d.Dist_settle 1.722615

11 d.Dist_water 1.545160

12 d.Aspect 1.375321

2 d.Forest 1.242597

5 d.Evi_min 1.240644

> d<-read.csv("Tiger Sample.csv",header=TRUE)

> d

Slope Forest Evi_Max Evi_mean Evi_min Evi_sd Dist_build Dist_road Dist_path Dist_settle

1 6.490818 45 0.858268 0.156967 0.047244 0.438207 0.007842 0.003345 0.063129 0.105994

2 9.280370 86 0.818898 0.232968 -0.732283 0.368572 0.058653 0.054411 0.096552 0.112101

3 3.738735 76 0.590551 0.102020 -0.448819 0.218236 0.020428 0.009338 0.066646 0.116286

4 19.466848 85 0.637795 0.219788 -0.362205 0.278388 0.038302 0.022240 0.077967 0.114316

5 1.530615 78 0.881890 0.369908 -0.456693 0.227464 0.028333 0.004969 0.048123 0.110956

6 5.254451 0 0.787402 0.033550 -0.551181 0.287994 0.060600 0.030067 0.088214 0.101296

7 3.019875 89 0.645669 0.175796 -0.425197 0.296354 0.054184 0.019185 0.076502 0.098337

8 2.353192 0 0.834646 0.257104 -0.409449 0.350769 0.026006 0.016008 0.029389 0.096890

9 11.362926 85 0.283465 -0.100822 -0.700787 0.298418 0.084361 0.051915 0.104129 0.102958

10 2.833498 32 0.708661 0.355871 -0.070866 0.174446 0.045677 0.000000 0.056405 0.097354

11 1.802090 0 0.921260 -0.084731 -0.488189 0.460455 0.054184 0.000786 0.056858 0.088698

12 7.488835 0 0.850394 0.071722 -0.417323 0.401299 0.072969 0.023534 0.084033 0.084500

13 1.037625 78 0.787402 0.257617 -0.015748 0.342238 0.033547 0.021663 0.034562 0.091844

14 20.297806 95 0.952756 -0.009757 -0.834646 0.465547 0.112258 0.067332 0.101781 0.102228

15 1.063464 66 0.944882 0.212770 -0.362205 0.344224 0.070608 0.015126 0.075664 0.080941

16 14.612314 22 0.929134 0.231941 -0.393701 0.426031 0.100131 0.053852 0.110278 0.093236

17 5.408870 47 0.771654 0.097056 -0.637795 0.356206 0.108171 0.059250 0.113300 0.091298

18 6.682181 0 0.937008 0.051866 -0.440945 0.347598 0.022030 0.007587 0.023963 0.077748

19 13.548040 0 0.921260 0.235193 -0.559055 0.378392 0.147699 0.076660 0.086987 0.063844

20 0.821789 78 0.937008 0.438548 -0.677165 0.276012 0.095169 0.041941 0.108960 0.077208

21 4.029274 70 0.566929 0.146183 -0.740157 0.237138 0.088108 0.027844 0.093652 0.069231

22 5.117507 67 0.921260 0.181616 -0.377953 0.328365 0.067429 0.003706 0.065808 0.063633

23 1.555901 78 0.929134 0.203013 -0.440945 0.461706 0.046136 0.015777 0.044806 0.067660

24 3.549428 74 0.842520 0.133516 -0.866142 0.464844 0.032530 0.009099 0.025333 0.065337

25 1.233278 78 0.937008 0.128381 -0.456693 0.329372 0.047496 0.023603 0.041205 0.055658

26 1.680633 69 0.433071 0.044163 -0.157480 0.159700 0.067798 0.003167 0.066225 0.048337

27 7.938294 78 0.803150 0.097056 0.015748 0.285248 0.069316 0.013278 0.085459 0.050344

28 0.973383 78 0.606299 0.239644 -0.275591 0.214753 0.023702 0.000278 0.017150 0.059146

29 8.708550 12 0.992126 0.415269 -0.480315 0.308960 0.077355 0.033148 0.108335 0.060732

30 10.210021 85 0.858268 0.310852 -0.251969 0.209354 0.103622 0.075008 0.125507 0.092256

31 3.811234 65 0.771654 0.246662 -0.267717 0.256779 0.052734 0.022788 0.046432 0.041690

32 10.958101 78 0.771654 0.265149 -0.133858 0.221819 0.084985 0.055079 0.132150 0.072683

33 3.518010 75 0.976378 0.474153 -0.094488 0.296725 0.052573 0.001389 0.076816 0.033084

34 4.001444 78 1.055118 -0.034406 -0.645669 0.358678 0.039210 0.009449 0.034998 0.049859

35 5.331814 78 0.866142 0.095344 -0.582677 0.405160 0.109084 0.085560 0.123101 0.090420

36 3.347971 69 0.181102 -0.099795 -0.732283 0.180848 0.029112 0.000000 0.027978 0.045597

37 1.192333 78 0.795276 0.100993 -0.346457 0.368253 0.062461 0.031353 0.112038 0.048688

38 4.552399 47 0.795276 0.098425 -0.527559 0.260358 0.012826 0.005996 0.015113 0.028335

39 1.677184 78 0.905512 0.312564 -0.716535 0.381764 0.048580 0.009821 0.091531 0.031124

40 11.066894 83 1.000000 0.364088 -0.370079 0.311637 0.150567 0.084757 0.085465 0.052693

41 33.361599 87 0.842520 0.173400 -0.614173 0.408416 0.137690 0.095533 0.097082 0.063617

42 5.381305 63 0.496063 0.089182 -0.393701 0.244549 0.027208 0.000278 0.029463 0.035573

43 11.904477 81 0.779528 0.190346 -0.606299 0.417857 0.072827 0.051220 0.133889 0.063735

44 6.687853 78 0.779528 0.322663 -0.070866 0.236124 0.055897 0.032558 0.116410 0.045156

45 8.032308 60 0.574803 -0.007874 -0.417323 0.256386 0.127603 0.103550 0.108629 0.075622

46 2.825631 78 0.661417 0.091236 -0.314961 0.243880 0.045192 0.000000 0.045933 0.045485

47 1.809811 32 0.826772 0.125300 -0.283465 0.456375 0.031013 0.001863 0.073498 0.012672

48 3.818700 0 0.748031 0.433927 -0.275591 0.170027 0.012273 0.008893 0.013517 0.017656

49 1.720547 78 0.779528 0.162787 -0.488189 0.331321 0.037812 0.013020 0.099018 0.024553

50 5.593223 85 0.716535 0.232797 -0.370079 0.255663 0.095415 0.077013 0.137896 0.089332

51 11.495823 0 0.881890 0.227662 -0.448819 0.396028 0.140954 0.078836 0.077940 0.051220

52 2.562000 66 0.543307 0.032352 -0.212598 0.417404 0.028232 0.010323 0.062932 0.023986

53 6.788340 70 0.866142 0.274735 -0.448819 0.379107 0.031877 0.004045 0.032103 0.036287

54 2.261904 71 0.858268 0.130264 -0.433071 0.367749 0.038145 0.005833 0.041867 0.033907

55 3.333536 70 0.811024 0.224752 -0.173228 0.297827 0.023282 0.009204 0.089471 0.017658

56 18.168600 78 0.519685 0.013009 -0.519685 0.271220 0.142217 0.101339 0.108407 0.077528

57 2.580121 0 0.842520 0.198733 -0.740157 0.379590 0.013162 0.011996 0.012251 0.017916

58 12.239436 47 0.834646 0.201301 -0.370079 0.309376 0.128863 0.090959 0.118048 0.086212

59 0.913544 13 0.653543 0.085758 -0.291339 0.244437 0.009167 0.008099 0.061677 0.015921

60 7.065469 75 0.905512 0.183156 -0.669291 0.392837 0.111660 0.077000 0.133086 0.100573

61 5.709737 78 0.622047 0.107669 -0.346457 0.249236 0.039114 0.020392 0.105845 0.037949

62 6.858332 62 0.503937 0.314790 -0.732283 0.149698 0.070344 0.050834 0.136369 0.068921

63 3.401820 66 0.913386 0.136255 -0.346457 0.325328 0.031449 0.009196 0.031822 0.019886

64 9.458386 70 0.795276 0.195310 -0.724409 0.409374 0.155016 0.103235 0.112771 0.085131

65 1.291215 78 0.732283 0.118110 -0.614173 0.377252 0.038523 0.000000 0.019405 0.020670

66 5.958159 87 0.897638 0.320781 -0.259843 0.309695 0.111439 0.068426 0.140641 0.108629

67 12.634109 0 0.905512 0.172544 0.047244 0.295580 0.125111 0.076894 0.132750 0.101721

68 7.498459 81 0.913386 0.109209 -0.314961 0.403591 0.153778 0.104980 0.103584 0.063035

69 5.617939 32 0.826772 0.236905 -0.338583 0.260162 0.018413 0.001944 0.007454 0.027245

70 3.222368 87 0.818898 0.171688 -0.763780 0.276739 0.041194 0.021791 0.104674 0.046696

71 6.344525 71 1.055118 0.362547 -0.299213 0.362756 0.167037 0.111942 0.110799 0.074330

72 16.689623 47 1.078740 0.159021 -0.488189 0.412735 0.053897 0.034321 0.117190 0.057909

73 10.226146 64 0.866142 0.332078 -0.401575 0.371824 0.083596 0.045962 0.145940 0.086255

74 1.376296 70 0.858268 0.243239 -0.244094 0.276906 0.032290 0.015126 0.093496 0.041915

75 5.544542 76 0.787402 0.130948 -0.488189 0.391938 0.153082 0.110177 0.108625 0.051101

76 2.909379 54 0.771654 0.150804 -0.385827 0.333480 0.167554 0.107320 0.121166 0.071117

Dist_water Aspect Elevation

1 0.002390 253.300751 197

2 0.013437 29.875992 1169

3 0.015280 284.743561 304

4 0.011554 270.000000 399

5 0.005727 234.462326 223

6 0.000000 184.398712 321

7 0.005457 273.814087 299

8 0.000000 275.194427 185

9 0.025593 273.814087 418

10 0.006961 299.054596 226

11 0.000000 146.309937 210

12 0.010808 320.194427 247

13 0.008722 18.434948 213

14 0.001496 159.775146 428

15 0.011142 170.537674 240

16 0.014022 147.200470 306

17 0.000621 79.508522 328

18 0.000878 4.635463 195

19 0.016574 138.179825 1156

20 0.013437 239.036240 278

21 0.023730 200.556046 237

22 0.027335 242.102737 230

23 0.013173 315.000000 208

24 0.017403 29.744881 204

25 0.023611 26.565050 206

26 0.022859 90.000000 219

27 0.013749 201.801407 214

28 0.018530 288.434937 201

29 0.000000 240.068497 243

30 0.001145 331.699249 323

31 0.029660 6.340191 202

32 0.001179 330.708649 263

33 0.004851 282.994629 216

34 0.033922 108.434952 197

35 0.003379 262.568604 416

36 0.021380 326.309937 188

37 0.004922 239.036240 236

38 0.005099 261.869904 164

39 0.006603 180.000000 216

40 0.006092 325.539185 884

41 0.000393 317.986633 528

42 0.009690 277.431396 188

43 0.008245 341.565063 293

44 0.003727 308.290161 240

45 0.002115 139.969742 493

46 0.024635 66.801407 194

47 0.007270 215.537674 194

48 0.000393 203.962494 160

49 0.005535 329.036255 212

50 0.000000 242.102737 334

51 0.000000 315.000000 583

52 0.025186 326.309937 197

53 0.005122 278.746155 183

54 0.018361 315.000000 191

55 0.001389 315.000000 204

56 0.001667 335.449554 418

57 0.000556 5.194428 164

58 0.001242 326.040924 333

59 0.009099 225.000000 185

60 0.009493 79.508522 391

61 0.010371 329.036255 235

62 0.015969 254.054611 331

63 0.021025 254.054611 184

64 0.018228 259.875336 428

65 0.003859 180.000000 183

66 0.013333 127.405357 360

67 0.000393 77.005386 298

68 0.001389 221.185928 401

69 0.001416 241.389542 168

70 0.001242 290.224854 217

71 0.002500 227.726318 385

72 0.001667 20.820892 251

73 0.026182 90.000000 346

74 0.009643 180.000000 199

75 0.000393 164.744888 349

76 0.007412 273.814087 340

[ reached 'max' / getOption("max.print") -- omitted 167 rows ]

> VIFcalc<-function(d)

+ {

+ result<-data.frame(var=c(names(d)),

+ VIF=numeric(length(d[1,])))

+ for(i in (1:length(d[1,])))

+ {

+ result$VIF[i] <-1/(1-summary(lm(d[,i] ~ .,data=d[,names(d)!=names(d)[i]]))$r.squared)

+ }

+ result<-result[sort(result$VIF,decreasing=T,index.return=T)$ix,]

+ return(result)

+ }

> VIFcalc(data.frame(d$Slope,d$Forest,d$Evi_Max,d$Evi_mean,d$Evi_min,d$Evi_sd,d$Dist_build,d$Dist_road,d$Dist_path,d$Dist_settle,d$Dist_water,d$Aspect,d$Elevation))

var VIF

7 d.Dist_build 3.882218

3 d.Evi_Max 3.205794

9 d.Dist_path 3.085667

8 d.Dist_road 2.473397

4 d.Evi_mean 2.257072

6 d.Evi_sd 2.215967

13 d.Elevation 1.897399

1 d.Slope 1.831618

10 d.Dist_settle 1.346418

5 d.Evi_min 1.336553

2 d.Forest 1.168984

11 d.Dist_water 1.155551

12 d.Aspect 1.113078
